# Vitamin K-dependent and other rare coagulation factor deficiencies: a single-center experience

**DOI:** 10.1186/s13052-025-02169-3

**Published:** 2025-12-08

**Authors:** Özlem Terzi, Sadık Sami Hatipoğlu

**Affiliations:** 1 Department Clinic of Pediatric Hematology and Oncology, Health Sciences University, Bakirkoy Dr. Sadi Konuk Training and Research Hospital, Istanbul, Turkey; 2 Department Clinic of Pediatrics, Health Sciences University, Bakirkoy Dr. Sadi Konuk Training and Research Hospital, Istanbul, Turkey

**Keywords:** Rare coagulation factor deficiency, Congenital coagulation disorders, Bleeding, Children, Treatment, Prophylaxis

## Abstract

**Background:**

Rare coagulation factor deficiency (RFD) is characterized by a deficiency of factor (F) I, FII, FV, FVII, FX, FXI, FXII, FXIII, or a combined deficiency of FV + FVIII or vitamin K-dependent factors and accounts for approximately 5% of all bleeding disorders. The prevalence of RFD in the general population can range from 1 in 1,000,000 for FX to 1 in 2–3 million for FXIII. Combined deficiencies of vitamin K-related factors have been reported in 30 families worldwide. These patients can present with a wide range of clinical symptoms, from mucocutaneous bleeding to life-threatening symptoms such as central nervous system and gastrointestinal bleeding. Treatment of these disorders is primarily based on the replacement of the deficient factor.

**Methods:**

In this retrospective study, data from 92 children with RFDs were analyzed to describe the distribution, clinical features, treatment patterns, and outcomes of RFDs.

**Results:**

The most common factor deficiencies were F VII and F XII deficiency and while combined vitamin-K dependent coagulation factor was found in 3 patients. Of the 92 patients included in the study, 72 exhibited bruising and/or bleeding. The most common type of bleeding was oral and nasal mucosal bleeding. Factor activity was ≤ 5% in 22 patients, 6–20% in 12 patients, and 20–50% in the remaining 60 patients. Among patients with factor levels < 5%, there were both patients without bleeding and patients with recurrent cerebral hemorrhage. Similarly, when factor levels reached 50%, some patients experienced bleeding while others remained asymptomatic. Acute and severe bleeding was controlled with treatment in nine patients. Twenty-seven patients with recurrent bleeding symptoms received prophylaxis. RFDs are more common in regions with high rates of consanguineous marriage, which was 29% in our study.

**Conclusions:**

No significant results were obtained regarding an increased risk of bleeding as factor plasmatic levels decreased in patients with RFD. Because of its autosomal recessive inheritance, improving access to genetic counseling and testing is important. Delays in diagnosis and treatment and lack of appropriate prevention are important risk factors that increase life-threatening bleeding.

## Background

Inherited factor deficiencies cause lifelong bleeding disorders. Rare factor deficiencies (RFDs) account for 3–5% of these bleeding disorders [1, 2]. The prevalence of RFDs worldwide ranges from 500,000 to 2 million [3]. Combined deficiencies of factors related to vitamin K have been identified in only 30 families in the world. Although they are very rare, they are important because they cause life-threatening blood loss [4]. However, they are most commonly encountered with mucosal hemorrhage.

Rare factor deficiencies consist of Factor I (Fibrinogen), Factor II (Prothrombin), Factor V, VII, X, XI, XII or XIII or combined Factor V and Factor VIII or combined factor deficiencies due to vitamin K (Factor II, VII, IX, X) [5]. Because RFDs are autosomal recessive, they can occur in both boys and girls. Therefore, in countries where consanguineous marriages are common, there are more cases of RFD than in Western countries.

Since RFDs are very rare and randomized controlled trials have not been performed, there are no evidence-based guidelines for treatment. Recommendations for treatment and prophylaxis are based on expert consensus [6].

If bleeding occurs, factor replacement (plasma-derived or recombinant) is administered; if unavailable, fresh frozen plasma (FFP) and cryoprecipitate are used. Prothrombin complex concentrate (PCC) and activated recombinant factor VII (rFVIIa) are other options. FFP contains all coagulation factors; cryoprecipitate contains fibrinogen, FVIII, FXIII and von willebrand factor; PCC contains FII, FVII, FIX, FX, protein C, protein S. Two new agents, recombinant FXIIIA and plasma-derived FX, have recently been added to the list of factors [7, 8]. Antifibrinolytic agents may be given in addition to factor replacement therapy to prevent mucosal hemorrhage [9].

In this study, we aimed to contribute to the management of these bleeding disorders by reviewing the laboratory and clinical features, treatment and prophylaxis approaches of patients with RFD followed in our center.

## Methods

### Patient selection

A total of 92 patients aged 0–18 years with any of the RFDs (F I, II, V, VII, X, XI, XII, XIII), combined deficiency of F V and F VIII and combined deficiency of vitamin K-related factors (F II-VII-IX-X) admitted to our Pediatric Hematology Outpatient Clinic in Istanbul, Turkey within the last 18 months were included in the study. Data were obtained from patient follow-up records in the electronic system. Patients’ presenting complaint, age at presentation, age at diagnosis, follow-up period, patients’ history of bleeding, consanguineous marriage between parents, family history of coagulation factor deficiency and/or bleeding, patients’ plasma factor plasmatic levels, treatment agents administered to patients receiving treatment and prophylaxis, and patients’ responses to these treatments were analysed.

Patients with plasma factor I, II, V, VII, X, X, XI, XII, XIII activity less than 50% were considered to have a rare coagulation factor deficiency. Abnormal tests were repeated twice to confirm the diagnosis. Mutation analysis could only be performed in 3 patients. The F X gene Next generation sequencing was requested in one patient, and the GGCX (gamma-glutamyl carboxylase) and VKOR (vitamin K 2,3-epoxide reductase) Next generation sequencing were requested in two patients with combined deficiencies of vitamin K-dependent factors.

Patients with rare coagulation factor deficiency who were also diagnosed with von Willebrand disease were excluded from the study to avoid misrepresentation of the laboratory and clinical features of RFDs.

This study was approved by the Ethics Committee of Bakirkoy Dr. Sadi Konuk Training and Research Hospital, Health Sciences University, Istanbul, Turkey. This study was conducted in accordance with the ethical standards as laid down of the 1964 Declaration of Helsinki. Written consent was obtained from the legal guardian parent of each participating child.

### Statistical analysis

Statistical description and comparison were performed using IBM SPSS, version 22 (IBM Inc., Armonk, NY, USA). Statistical analysis was performed utilizing Fisher’s exact or t-test. Logistic regression was used to generate odds ratios and 95% confidence intervals. p-values < 0.05 were considered statistically significant. Significance was evaluated at the *p* < 0.05 level.

## Results

### Patient characteristics

Of the 92 rare coagulation factor deficiency patients, 47 were female. The median age at symptom onset was 9 years (range 0–17). Of all patients, 4 had a family history of rare coagulation factor deficiencies, 23 had a family history of bleeding/bruising, and 27 had consanguineous marriage between parents. The diagnosed factor deficiencies were FI (*n* = 15), FV (*n* = 5), FV + FVIII deficiency (*n* = 2), FVII (*n* = 21), FX (*n* = 10), FXI (*n* = 7), FXII (*n* = 22), FXIII (*n* = 7), vitamin K-dependent combined coagulation factor deficiency (*n* = 3) (Table 1).

**Table 1:** The demographic and clinical features of our patients

	*n* (%)
Age (median) (years)	9 (0–17)
Sex	
• Female	47 (51)
• Male	45 (49)
Consanguinity	
• Present	27 (29)
• Absent	65 (71)
Family History	
• Present	23 (25)
• Absent	69 (75)
Bleeding/ bruising symptoms	
• Symptomatic	72 (78)
• Asymptomatic	20 (22)
Factor (F) deficiency	
• F I deficiency	15 (16)
• F II deficiency	0
• F V deficiency	5 (5)
• F V + VIII deficiency	2 (2)
• F VII deficiency	21 (23)
• F X deficiency	10 (11)
• F XI deficiency	7 (8)
• F XII deficiency	22 (24)
• F XIII deficiency	7 (8)
• Vitamin K-Related Combined Factor Deficiencies	3 (3)

### Bleeding symptoms

Bruising and/or bleeding were observed in 72 of 92 patients included in the study. Of all patients, 51 had a history of hemorrhage but no bruising, 7 had both bleeding and bruising, and 14 had only bruising. Bleeding or bruising was not observed in 20 (22%) of all patients; two of these patients came for examination because their fathers had coagulation factor deficiency, and 18 were referred to hematology because of high PT or PTT values obtained preoperatively or incidentally. Bleeding sites according to coagulation factor deficiency are shown in Table 2.

**Table 2:** The distribution of sites of bleeding symptoms by coagulation factor deficiency

Symptoms	Coagulation Factor Deficiency (*n*)									
	FI	FII	FV	FV + FVIII	FVII	FX	FXI	FXII	FXIII	vK CFD
Mucodermal bleeding	-	-	1	-	2	1	1	3	-	2
Epistaxis	5	-	1	1	10	6	1	6	1	-
Menstrual bleeding	3	-	1	-	1	1	2	10	4	-
Circumcision bleeding	-	-	2	1	1	1	-	1	-	-
Vaginal bleeding	-	-	-	-	-	1	-	-	-	-
İntracranial bleeding	1	-	-	-	1	1	-	-	-	-
Skin bleeding	-	-	-	-	-	-	-	-	-	2
Gastrointestinal bleeding	-	-	-	-	-	-	-	-	-	1
bruise on the skin	3	-	2	-	5	-	1	4	2	-
bleeding after injection	-	-	-	-	-	-	1	-	1	-
hematoma (in the joints)	-	-	-	-	1	-	-	-	-	-
no bleeding and bruise	3	-	1	1	6	3	4	5	1	1

N: number of patients

### Factor plasmatic levels

Factor activity was ≤ 5% in 22 patients, 6–20% in 12 patients, and 20–50% in the remaining 60 patients. Factor activity levels (%) according to coagulation factor deficiency are shown in Table 3.

**Table 3:** Factor activity (%) by coagulation factor deficiency in 92 patients with rare coagulation factor deficiency

Factors deficiency	Factor activity (%)		
	≤ 5	6–20	21–50
F I deficiency	15	-	-
F II deficiency	-	-	-
F V deficiency	-	-	5
F V + VIII deficiency *	2	2	-
F VII deficiency	-	2	19
F X deficiency	1	0	9
F XI deficiency	1	0	6
F XII deficiency	1	6	15
F XIII deficiency	-	1	6
Vitamin K-Related Combined Factor	2	1	-

N: number of patients

* F VIII levels were < 5% in both patients, while F V levels were 11% and 12%

### Bleeding symptoms by factor plasmatic level

In F I deficiency, factor plasmatic levels were between 1.2 and 1.4% (< 5%). Despite the same factor plasmatic levels, bleeding/bruising was not observed in 3 of 15 patients, whereas life-threatening recurrent cerebral hemorrhage was observed in 1 patient. Factor plasmatic levels of 5 children with factor V deficiency were between 28 and 44%. Despite similar factor plasmatic levels, there were patients with a history of mucosal bleeding and bleeding that did not stop after circumcision as well as patients with no history of bleeding. In combined factor V and FVIII deficiency, FV and FVIII levels were 11%-4% and 12%-1%, respectively. The first patient had a history of bleeding that did not stop after circumcision and recurrent epistaxis was detected during follow-up; the other patient had no bleeding. In 15 of the 21 cases with factor VII deficiency, plasma factor levels ranged from 6% to 48%, and epistaxis, bruising, intracranial hemorrhage, or bleeding that did not stop after circumcision was observed. In the other 6 cases with FVII deficiency, factor plasmatic levels were between 19 and 45% and no bleeding was observed. In 9 of 10 patients with factor X deficiency, factor plasmatic levels were between 24 and 44% and epistaxis/menorrhagia was observed in 6, and no bleeding was observed in 3. Recurrent life-threatening bleeding (cranial, vaginal, nasal) was observed in a 2-month-old girl with a factor plasmatic level < 1%.

In 6 of the 7 patients with FXI deficiency, factor plasmatic levels were between 23 and 47% and bleeding (menorrhagia, bruising, mucosal bleeding) was observed in 4 but not in 2 patients. In 1 patient, the factor plasmatic level was 4% and there was no history of bleeding. In 2 of 22 patients with FXII deficiency, factor levels were 3% and 6%, respectively, and both had epistaxis. Children with a factor plasmatic level of 8% had no history of bleeding. Factor plasmatic levels were between 17 and 50% in those without bleeding. In factor XIII deficiency (n. 7 patients), factor plasmatic levels ranged between 14 and 50%, and bleeding (menorrhagia, bruising, bleeding after injection) was observed in 6 of them. Bleeding (skin, gastrointestinal and oral mucosal bleeding) was observed in 2 patients with vitamin K-dependent coagulation factor plasmatic deficiency and factor plasmatic levels < 20%, while bleeding was not observed in the other patient with the same factor plasmatic levels.

### Treatment and prophylaxis

For treatment, among patients with recurrent and/or non-stop bleeding, 4 patients received fresh frozen plasma (FFP) alone; 2 received FFP and recombinant coagulation factor VIIa; 1 received FFP, PCC and recombinant coagulation factor VIIa; 2 received vitamin K. For prophylaxis, 1 received only FFP; 1 received only PCC; 1 received FFP and fibrinogen concentrate; 1 received PCC and an antifibrinolytic agent; 2 received only vitamin K; and 19 received only an antifibrinolytic agent (tranexamic acid) (Table 4).

**Table 4:** Distribution of patients’management according to coagulation factor deficiency

Coagulation Factor deficiency	Treatment (*n*)	Prophylaxis (*n*)
F I	FFP (1)	FFP and fibrinogen concentrate (1), tranexamic acid (2)
F II	-	-
F V	FFP (2)	tranexamic acid (1)
F V + VIII	-	-
F VII	FFP and rF7a (2)	tranexamic acid (4), rF7a (2)
F X	FFP (1)	FFP (1), PCC (1), tranexamic acid (3)
F XI	-	tranexamic acid (1)
F XII	-	tranexamic acid (5)
F XIII	-	tranexamic acid (3)
Vitamin K-Related Combined Factor	FFP/Vitamin K/PCC/tranexamic acid/ recombinant factor 7a(1); Vitamin K(2)	PCC and tranexamic acid (1), vitamin K (2)

N: number of patients; RF7a: Recombinant factor 7a

Two brothers with factor V deficiency were treated with FFP for persistent bleeding after circumcision. One of the patients received tranexamic acid prophylaxis for recurrent gingival haemorrhage during more than ten years of follow-up. One patient with FVII deficiency with bleeding after circumcision and head trauma and 1 patient with joint hematoma were treated with FFP and rF7a (recombinant Factor 7a) and given rF7a prophylaxis; 4 patients with FVII deficiency received tranexamic acid prophylaxis.

A patient with factor X deficiency had a factor plasmatic level of 34% and was receiving PCC prophylaxis for severe menorrhagia and anaemia (Hb:5 g/dL). Another two-month-old female patient with factor X deficiency experienced three life-threatening bleeding episodes (intracranial hemorrhage, vaginal hemorrhage, and epistaxis) and she received FFP treatment. After the first 2 bleeding episodes, prophylaxis was not initiated at an external centre; no bleeding was observed after FFP prophylaxis was initiated. F X gene (c.785G > A) sequence analysis was positive in this patient. Patients with recurrent epistaxis or menorrhagia and factor XI, XII or XIII deficiency were also started on tranexamic acid prophylaxis.

Our first patient with vitamin K-dependent combined coagulation factor deficiency began experiencing bleeding in the oral mucosa at 4 months of age and has been receiving vitamin K prophylaxis ever since; prophylaxis cannot be discontinued because mucosal bleeding recurs when it is stopped. At initial diagnosis, vitamin K–dependent coagulation factors were found to be below 5%, and genetic testing revealed a *GGCX mutation* (c.44–57 > A, p.(?), chr2:85788113) through sequence analysis, as well as a *VKORC1 mutation* (*c.-1639G > A*, rs9923231) and CYP2C9*3 (p.I359L, rs1057910) positivity. The second patient with vitamin K-related combined coagulation factor deficiency had been experiencing mild to moderate skin and gum bleeding since the age of 2 and was not receiving prophylaxis. The patient experienced two episodes of severe gastrointestinal bleeding at the age of 16. The patient, who did not benefit from vitamin K and developed anaphylaxis to FFP, was treated with PCC and rFVIIa. The patient received PCC and antifibrinolytic prophylaxis twice a week and did not experience recurrent severe bleeding. *GGCX* sequence analysis and *VKOR* genetic screening results were negative. The third patient, who had the same diagnosis and plasma factor levels of 20%, had never experienced bleeding throughout his life.

## Discussion

RFDs are a heterogeneous group of inherited coagulation factor deficiencies with different clinical manifestations. Among RFDs, factor VII and XI deficiencies are the most common [10]. According to the results of our study, the most prevalent RFDs were deficiencies in factors VII and XII. Haemophilia is the most frequent hereditary coagulation factor deficiency, and extensive research has been conducted due to the high number of cases, resulting in the preparation of evidence-based guidelines. However, since RFDs are very rare, studies are limited and there are no guidelines for the management of these patients. The management of RFDs is based on expert consensus.

Hematomas and intramuscular bleeding are prevalent among patients with severe hemophilia. The most typical type of bleeding associated with RFD is mucocutaneous bleeding. In our study, a hematoma was observed in only one patient, and the most frequent types of bleeding were epistaxis and gingival bleeding. However, life-threatening hemorrhages can occur in individuals with RFD [11, 12]. Of our cases, one involved severe gastrointestinal bleeding and three involved intracranial bleeding.

Determining a clear factor plasmatic level at which the likelihood of bleeding increases is the greatest challenge in the management of RFDs. It has been reported that the risk of haemorrhage increases when the fibrinogen level is < 50 mg/dL (< 0.5 g/L) [13]. In our study, the F1 levels of our 15 patients ranged between 1.2 and 1.5 g/L. While 3 cases with similar factor plasmatic levels were asymptomatic, the others had a history of haemorrhage/ecchymosis, and 1 patient even had cerebral haemorrhage twice. In factor V deficiency, it has been reported that bleeding is not observed when the factor plasmatic level is >10% [14]. In our study, the F V level of 5 patients was between 29% and 44% and 1 patient was asymptomatic and 4 had a history of bleeding/purpling. Studies have reported different results between factor VII level and severity of bleeding [15, 16]. The recommended factor plasmatic levels for no haemorrhage vary as >10%, >20% and >1% [14, 17]. In our study, recurrent hematomas were observed in the joints of 1 patient and the factor plasmatic level was 6%. In the other 20 children with factor VII deficiency, factor plasmatic levels ranged between 19 and 48% and 6 of them were asymptomatic. Studies have shown that the minimum FX level required for patients to remain asymptomatic may range from 10% to 40% [18]. In our study, our patient with an FX level of < 1% presented with recurrent life-threatening bleeding, while our patient with a factor plasmatic level of 34% was receiving prophylactic treatment for severe menorrhagia. Severe bleeding has been reported when the level is < 20% in FXI deficiency and < 30% in FXIII deficiency [14]. In our study, the patient with a factor FXI level of 4% was asymptomatic. The patient with an F XI level of 47% had a history of persistent bleeding after intramuscular injection. In FXII deficiency, bleeding is not an expected finding, including during major surgery; skin bruising and nosebleeds have rarely been reported. In our study, 17 of 22 patients with FXII deficiency had a history of bleeding/ecchymosis. Despite similar factor plasmatic levels, one of the 3 children with vitamin K-dependent combined coagulation factor deficiency was asymptomatic, while one had mucosal bleeding and the other had life-threatening bleeding. In a study conducted on 489 patients with RFD, the relationship between factor plasmatic levels and bleeding was investigated [14]. According to the results of the study, a strong relationship was found between F I, combined F V and VIII, F X and F XIII levels and bleeding; a weak relationship was found for F V and F VII, and no relationship was found for FXI. The data in our study suggest that there is no relationship between factor plasmatic levels and the likelihood of bleeding, in accordance with the literature.

There are molecular studies showing correlations between gene mutation and absence of bleeding symptoms. One study reported a p.R462X mutation explaining asymptomatic FVII deficiency and the role of the variable carboxy terminus of coagulation serine proteases [19]. Factor VII deficiency is the most common among rare inherited autosomal recessive bleeding disorders, and is a chameleon disease due to the lack of a direct correlation between plasma levels of coagulation Factor VII and bleeding manifestations [20].

The differences observed among present patients may be, then, due to genetic and/or environmental factors. Various studies have reported the rate of consanguineous marriages among parents of children with RFD to be between 48.6% and 65% [17, 21, 22]. In our study, this rate was found to be 29%. Where bleeding disorders and consanguineous marriages are common, family screening and genetic counselling are recommended to prevent major complications and improve patients’ quality of life. Studies emphasize that the quality of life of children with bleeding disorders and their families can be improved through both medical and non-medical (psychological) interventions [23]. The role of genetic research in advancing (and in some cases determining) genotype-phenotype correlations and subsequently providing more accurate prognostic assessments in addition to risk of recurrence and/or pre-conception/prenatal diagnosis (primary and secondary prevention) is noteworthy. Although the factor level associated with severe bleeding in patients with F VII deficiency was determined to be 7.5%, it was emphasized that broader cohort data containing information on environmental and genetic factors is needed [24]. In another study, it was reported that F7 genotypes (rs510335, rs5742910, rs561241, rs36209567, and rs6046) showed a weak correlation with the severity of clinical symptoms, but a strong correlation with FVII levels [25]. Although vitamin K-dependent coagulation factor deficiency is known to be molecularly associated with mutations in the GGCX or VKORC1 genes, one study indicated that the VKORC1-wildtype type increases the risk of intraventricular hemorrhage in infants [26, 27]. The identification of new mutations can provide useful information about the genetic basis of the disease by contributing to the evaluation of genotype/phenotype correlations, and this approach may enable the primary prevention of genetic diseases in the future.

The traditional management of RFDs is to treat bleeding as it occurs. It is important to administer the correct treatment at the appropriate dose and in a timely manner. Inadequate replacement therapy in F X deficiency has been reported to cause approximately 50% of all deaths [28]. Prophylaxis is recommended in patients with life-threatening bleeding and in cases of recurrent bleeding. Fibrinogen, FXIII, and FVII concentrate are available in daily clinical activity. Prothrombin, and FV concentrate are not available. A new FV concentrate is being developed for clinical use [7, 8]. Plasma-derived FX is routinely used in Europe and the USA, but is not available in our country. If factor concentrate is not available, FFP, tranexamic acid, PCC and rF7a are used for treatment and prophylaxis. In our study, when factor concentrate was not available in cases with severe bleeding, treatment with FFP, PCC and rF7a was administered and bleeding control was achieved in all patients. No new major bleeding was observed in patients receiving prophylaxis. Prophylaxis appears to be an important and useful option in cases with a history of major bleeding.The limitations of our study are the retrospective nature of the study, the small size of the cohort, and the fact that genetic mutation analysis was not performed in all patients.

The limitations of our study include its retrospective design, the small cohort size, and the fact that genetic analysis was performed in only a few patients.

## Conclusion

Combined deficiency of RFDs and vitamin K–dependent factors is very rare, and studies on this topic are limited, with our 92 single-center cases representing a significant sample. RFDs may be asymptomatic or can lead to life-threatening bleeding. Because the number of cases is small, it remains unclear how to identify patients at risk of severe bleeding. There is a lack of correlation between plasma factor levels and the severity of clinical manifestations. Evaluation of the genetic profile may provide useful support for clinicians, particularly in identifying individuals who might benefit from prophylaxis.

## Data Availability

The data generated during the current study are available from the corresponding author upon reasonable request.
